# An optimized base editor with efficient C-to-T base editing in zebrafish

**DOI:** 10.1186/s12915-020-00923-z

**Published:** 2020-12-03

**Authors:** Yu Zhao, Dantong Shang, Ruhong Ying, Hanhua Cheng, Rongjia Zhou

**Affiliations:** 1 Hubei Key Laboratory of Cell Homeostasis, College of Life Sciences, Wuhan University, Wuhan, 430072 China; 2 Renmin Hospital of Wuhan University, Wuhan University, Wuhan, 430072 China

**Keywords:** CRISPR/Cas9, Base editor, BE4max, Zebrafish

## Abstract

**Background:**

Zebrafish is a model organism widely used for the understanding of gene function, including the fundamental basis of human disease, enabled by the presence in its genome of a high number of orthologs to human genes. CRISPR/Cas9 and next-generation gene-editing techniques using cytidine deaminase fused with Cas9 nickase provide fast and efficient tools able to induce sequence-specific single base mutations in various organisms and have also been used to generate genetically modified zebrafish for modeling pathogenic mutations. However, the editing efficiency in zebrafish of currently available base editors is lower than other model organisms, frequently inducing indel formation, which limits the applicability of these tools and calls for the search of more accurate and efficient editors.

**Results:**

Here, we generated a new base editor (zAncBE4max) with a length of 5560 bp following a strategy based on the optimization of codon preference in zebrafish. Our new editor effectively created C-to-T base substitution while maintaining a high product purity at multiple target sites. Moreover, zAncBE4max successfully generated the Twist2 p.E78K mutation in zebrafish, recapitulating pathological features of human ablepharon macrostomia syndrome (AMS).

**Conclusions:**

Overall, the zAncBE4max system provides a promising tool to perform efficient base editing in zebrafish and enhances its capacity to precisely model human diseases.

## Background

A large number of human genetic diseases are caused by point mutations or disease-associated SNP variants [1]. Large-scale genome-wide association studies have identified many loci that are associated with key human traits and diseases, for example, lifespan and aging [2]. In recent years, deep whole-genome sequencing of human populations generated numerous databases of genetic variations, including SNPs of pathogenic variants. Mutation spectrum analysis indicated that major mutations were C:G>T:A and T:A>C:G transitions associated with metabolic traits and disorders [3]. Although some of these mutations have been determined as causal or pathogenic variants, many disease-associated SNP variants remain to be defined by functional investigations.

Zebrafish has become a popular model species for the study of gene function [4, 5]. Genome sequencing showed that approximately 70% of human genes have at least one obvious zebrafish ortholog [5]. Gene mutations in zebrafish can indeed mimic many human diseases, for example, ATP-sensitive potassium channel mutation for genetic cardiovascular disorder [6] and *otx2b* mutation for endocrinal disorder [7]. CRISPR/Cas9 and next-generation gene-editing techniques provide a fast and efficient tool for making genetically modified zebrafish for modeling pathogenic mutations in humans.

Inducing single-nucleotide substitutions in animal genomes can bring great advantages to studies of disease modeling and gene therapy [8]. Recently, base editing is a developing approach that can efficiently introduce point mutations in a programmable way without inducing double-strand breaks (DSBs) [9]. Until now, two classes of DNA base editor—cytosine base editor (CBE) and adenine base editor (ABE)—have been described and widely used in plants and animals, including zebrafish [10–14]. In addition, several strategies were developed to expand the base editors, such as a narrowed editing window, expanded targeting scope [15], improved editing specificity [16], and changed protospacer adjacent motif (PAM) compatibilities [15]. Although CBE exhibits high editing efficiency in mammalian cells, only a limited ability was reported in zebrafish [13, 17]. BE3 system can induce base conversion efficiently in zebrafish [13]. However, compared with other model organisms, the base substitution efficiency is a bit low and frequently induces indel formation, which greatly limits the applicability of the tool in zebrafish. Previous studies have demonstrated that an optimized cytidine base editor, BE4max, modified by nuclear localization signals and codon usage, and ancestral reconstruction of the deaminase component can improve editing efficiency in human cells [16]. Therefore, we attempted the strategy to optimize BE4 in zebrafish. We optimized codon usage in BE4max editor for better expression in zebrafish and synthesized whole components for the editor. The final optimized zAncBE4max with a length of 5560 bp was a construct with complete “zebrafish” genes. The codon-optimized zAncBE4max showed a high editing efficiency at multiple target sites. We further used the base editor to generate C-to-T base conversion in *twist2*, and the zebrafish mutant recapitulated pathological features of ablepharon macrostomia syndrome (AMS) in humans. Thus, this study provides a new tool for precise genome editing and enriches the base editing toolkit in zebrafish.

## Results

### zAncBE4max system induces C-to-T base conversion efficiently in zebrafish

**Fig. 1:**
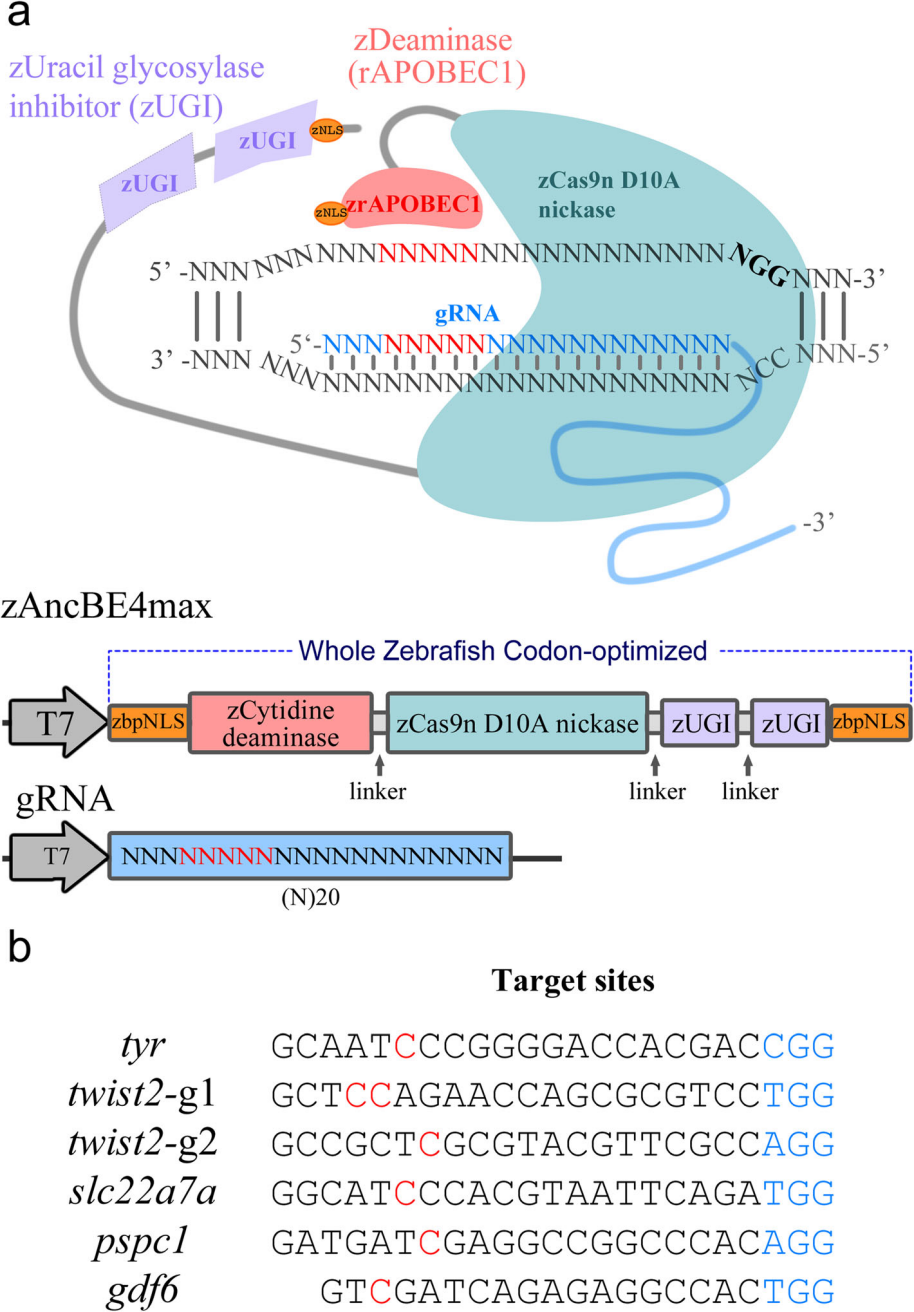
Base editor in zebrafish using a whole zebrafish codon-optimized BE4 system. **a** Schematic diagram of the zAncBE4max system. The system consists of whole zebrafish codon-optimized genes/components, including rat deaminase APOBEC1(red), Cas9n D10A nickase (green), two uracil DNA glycosylase inhibitors (purple), and a bipartite NLS at both ends, which convert cytidine to thymine within a window of − 17 to − 13 bases from the PAM of the gRNA. Both zAncBE4max and gRNA are linked to the T7 promoter for in vitro transcription. Prefix “z” indicates zebrafish codon-optimized components. **b** The targeted genes and corresponding sites designed and used in this study. PAM region (blue); target cytidines (red)

Zebrafish codon-optimized C-to-T base editor included cytidine deaminase with an ancestral edition (Anc689), Cas9n D10A nickase, and two UGIs in tandem at the C termini, which was flanked by bipartite NLS sequences at both N- and C-termini for nuclear localization. To optimize their expression in zebrafish, we changed the nucleotide bases according to zebrafish codon preference, GC content, and secondary structure, and synthesized these components, based on original components of AncBE4max [16]. The final optimized zAncBE4max with a length of 5560 bp had complete “zebrafish” genes (Fig. 1a; Additional file 1: Fig. S1). We designed guide RNAs (gRNAs), taking into account the following factors (Fig. 1b). Considering the very high polymorphisms in the zebrafish genome, we first determined the target sequences in the genome. The system zAncBE4max has a cytidine deaminase fused to Cas9 nickase (nCas9) that mediates the direct conversion of C to T in zebrafish. The selected gRNA needs to have the targeted C covered in the specific target region. The optimal deamination sites for the system are located in a 5-bp window on the target site and − 17 to − 13 bp upstream of the PAM sequence.

**Fig. 2:**
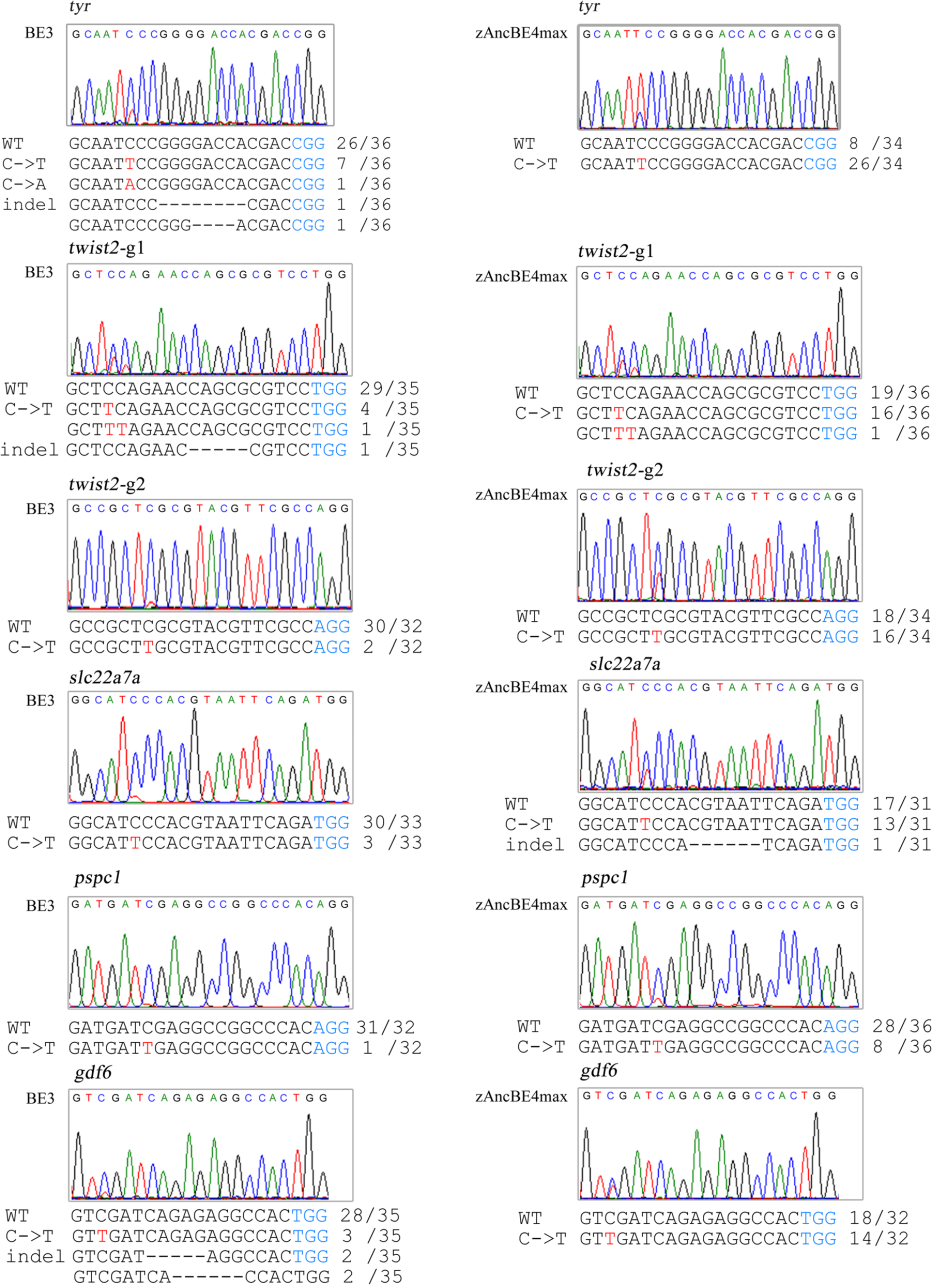
Base C-to-T editing efficiency using zAncBE4max in zebrafish. The zAncBE4max system (right panel) showed higher activity of C-to-T editing than the BE3 system (left panel) at 6 tested sites. Red letter C indicates sites of C-to-T conversion and corresponding overlapped peaks in sequencing chromatogram. PAM region is indicated in blue. Mutant number in sequenced clones is shown on the right of sequences

A previous study has demonstrated that the zebrafish *tyr* locus can be edited by the BE3 system [13], so we first chose the same locus to test the zAncBE4max system’s base editing ability. After injecting the zAncBE4max mRNA and related *tyr* gRNA into the embryos of one-cell stage, 6 embryos that developed normally were randomly selected and mixed together for genomic DNA extraction at 48 h post-fertilization (hpf). In comparison with BE3, the overlapping peaks at the targeted cytidine at the *tyr* locus were indeed higher in the zAncBE4max system by sequencing the PCR results. Further T-A cloning proved that zAncBE4max showed a higher activity than BE3 in the target site (Fig. 2).

Next, we evaluated zAncBE4max at another 5 targets (*twist2-g1*, *twist2-g2*, *pspc1*, *slc22a7a*, and *gdf6*) to determine C-to-T base editing. After embryo microinjection, sequencing of these target products showed that zAncBE4max improved editing efficiency compared to BE3 at rates over 3-fold at these targets (Fig. 2). In addition, the product purities of zAncBE4max (ratios of desired point mutations to indels and undesired mutations at the target nucleotide) were better than those of BE3 (Fig. 2), suggesting that the zAncBE4max system efficiently induced C-to-T base editing with the high product purity in zebrafish. To examine the germline transmission ability of the edited targets, F_1_ generation was obtained by F_0_ crossing with wild type, and the number of zebrafish with the edited target in F_1_ was counted, which showed the average rate of germline transmission higher in zAncBE4max than in BE3 (50.61% vs. 20.24%)(Additional file 2: Table S1). These data indicated that the zAncBE4max system efficiently induced C-to-T base editing with high germline transmission in zebrafish.

**Fig. 3:**
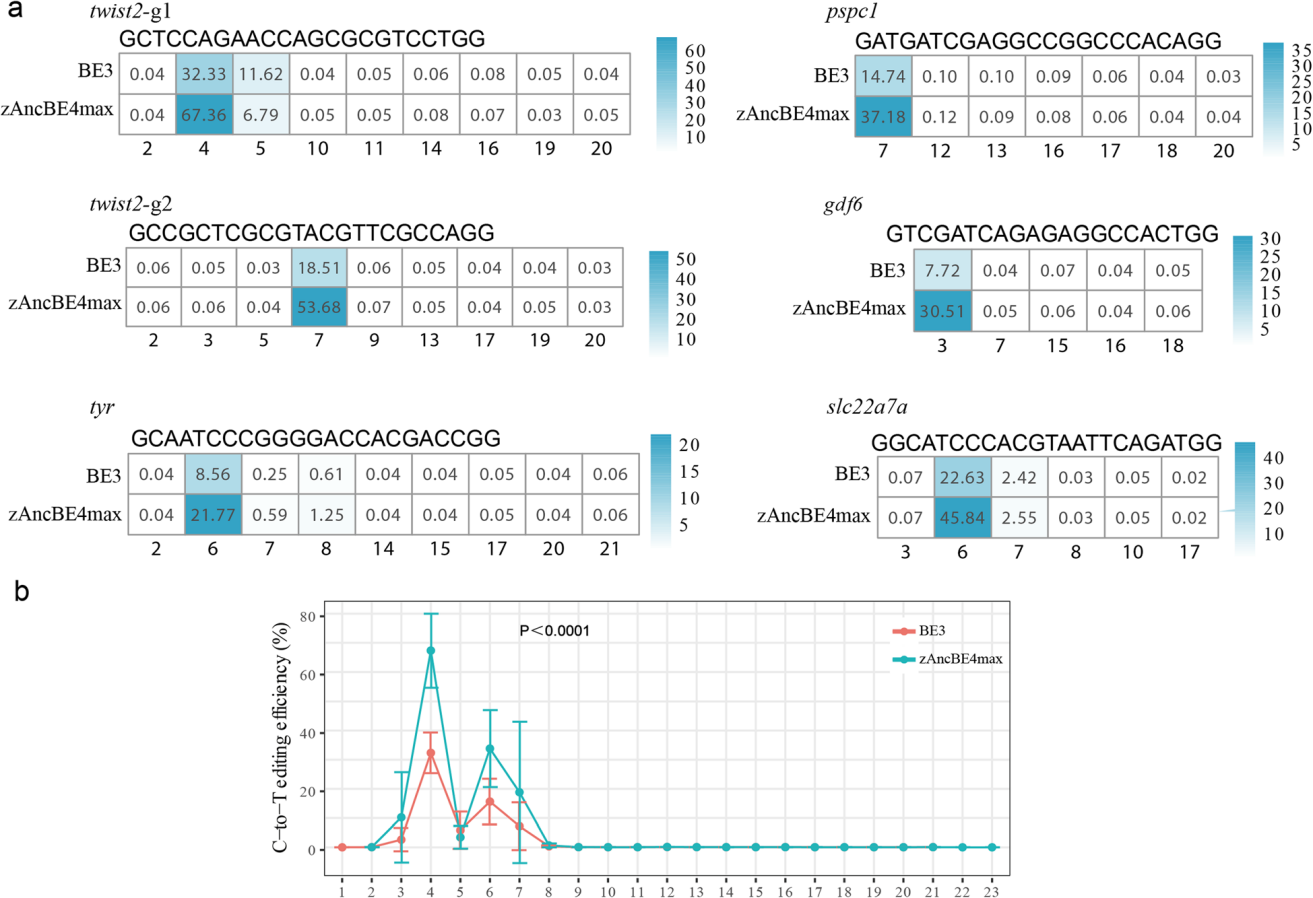
The on-target C-to-T editing efficiency of BE3 and zAncBE4max at 6 target sites. **a** Percentage of each C-to-T conversion along the target sequence for comparison of BE3 and zAncBE4max. **b** Overall C-to-T editing efficiency for BE3 and zAncBE4max at each C of the 6 target sites. Mann-Whitney *U* test was performed. *n* = 3 biologically independent samples for each target

To characterize the C-to-T base editing system in detail, we performed deep sequencing of the 6 target sites using the MGISEQ2000 sequencing platform. Genomic DNA from a pool of 100 injected embryos for each target site with 3 injection repeats was used for PCR amplification, and an amplicon library was constructed for each target. After sequencing and alignment with wild-type sequences, 118,427~24,564,283 reads were obtained for a target site (Additional file 2: Table S2). The average rate of C-to-T conversion of these 6 targets in zAncBE4max was 42.723% with the highest rate of 67.36% in *twist2*; in contrast, the average value was 17.415% in BE3 (Fig. 3a; Additional file 2: Table S3), confirming that the zAncBE4max system induced C-to-T base editing efficiently.

Notably, the optimal C-to-T editing sites in the 5-bp window were nt 3–7 on the target with the highest level at nt 4 and nt 6 (Fig. 3b). In addition, indel generation was lower in zAncBE4max than in BE3 (0.288% vs. 1.2%) (Additional file 1: Fig. S2). Together, these data suggested that the zAncBE4max system can induce C-to-T base conversion efficiently in the zebrafish genome.

### Modeling pathogenic mutations using the optimized base editor

**Fig. 4:**
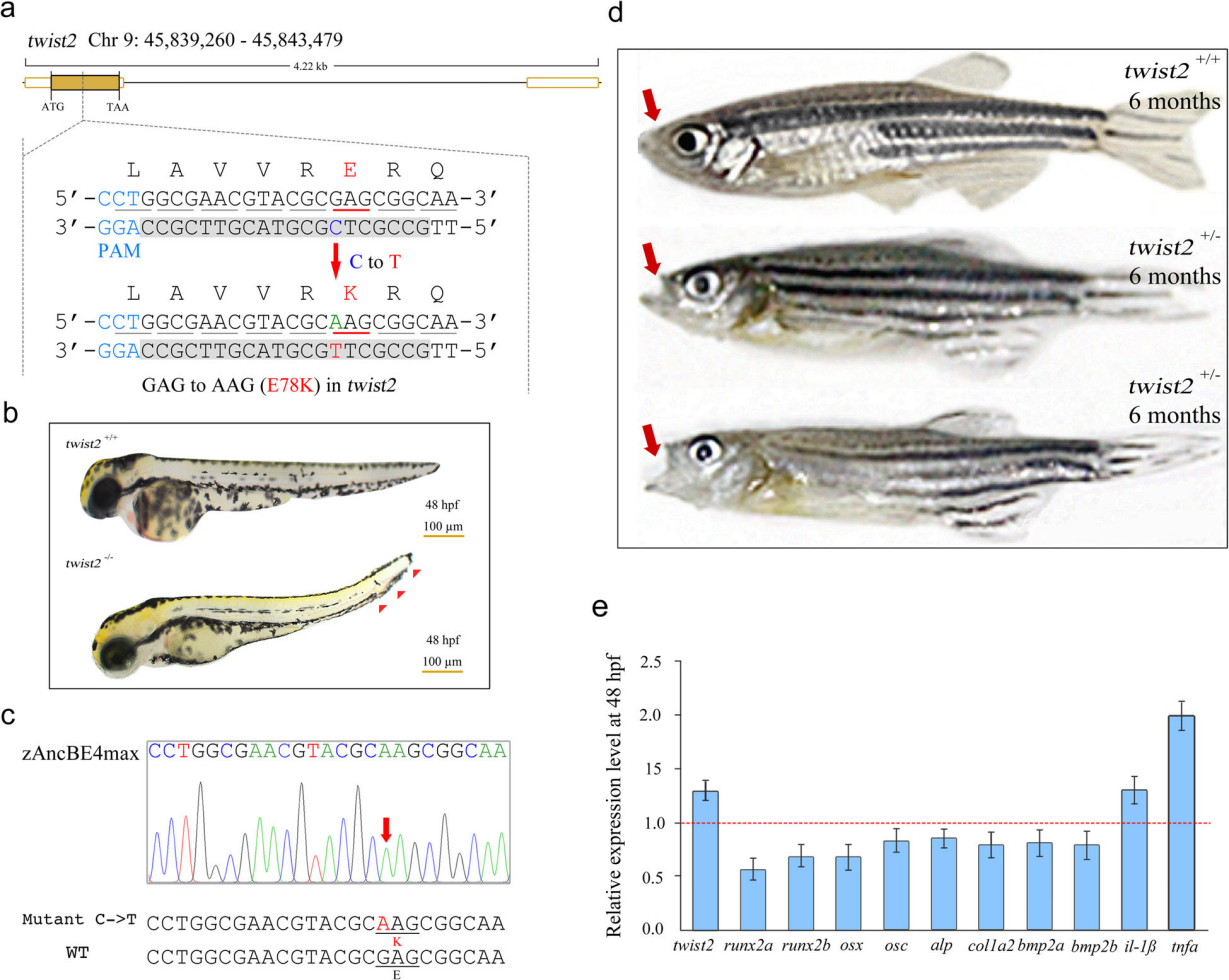
Generation of an AMS zebrafish model using the zAncBE4max system. **a** The designed target site in the *twist2* gene on chromosome 9 in zebrafish. The nucleotide changes in C-to-T editing are underlined in red. The corresponding amino acid change is E78K. PAM region is indicated in blue. **b** Homozygous mutant (E78K) embryos exhibit a short truck and curved tail (red arrowheads) at 48 hpf. Scale bar, 100 μm. **c** Genotyping of homozygous mutant larva by PCR and sequencing. Red arrow indicates point mutation site C-to-T. **d** Phenotype of heterozygous mutants (E78K) exhibited a protruding jaw, unclosed mouth (red arrows), and emaciated body at the age of 6 months. For more details, see Additional file 3: Movie 1. **e** qRT-PCR analysis indicates the relative expression levels of *twist2* and related genes in *twist2* homozygous E78K mutant embryos

Ablepharon macrostomia syndrome (AMS) is one kind of congenital ectodermal dysplasia, characterized by absent eyelids, microtia, redundant skin, variable abnormalities of the nipples, and poor growth [18]. Clinical studies and whole-exome sequencing showed that *twist2* is the causative gene for AMS [19]. We want to investigate the ability of the zAncBE4max system to build the model in zebrafish. The zAncBE4max system was used to target at the *twist2* (E78K) locus (Fig. 4a). Further breeding and mutant analysis showed that homozygous E78K mutants exhibited a phenotype of short truck and curved tail at 48 hpf and died at about 15 dpf with unknown reasons (Fig. 4b,c). Since AMS is an autosomal-dominant fashion in humans, we focus on observing the phenotype of heterozygous larva. Interestingly, heterozygous mutants can survive to adults and be fertile, but most heterozygous adults showed the phenotypes of protruding jaw, unclosed mouth, and emaciated body at about 6 months (Fig. 4d; Additional file 3: Movie 1), which mimics the human phenotypes with pronounced face abnormalities.

Previous studies have shown that the E75K mutation in *twsit2* altered the DNA binding activity of itself, leading to both gain of function and dominant-negative effects. Twist2 regulated cytokine expression through a negative feedback loop that repressed NF-κB activity and was highly expressed in the craniofacial mesenchyme and chondrogenic precursors [19, 20]. We further examined the expression of *twsit2* and related pathway genes. Quantitative RT-PCR showed that *twsit2* itself expression was indeed increased in homozygous embryos, and the proinflammatory cytokines, *Il-1β* and *tnfa*, were found highly expressed. In addition, the expression of the genes involved in bone development was decreased (Fig. 4e), which is consistent with the previous study. Overall, these data suggested that the engineered zAncBE4max variant successfully induced C-to-T point mutation of *twist2* in zebrafish, which greatly recapitulate pathological features of human AMS.

## Discussion

The optimized zAncBE4max system presented here obviously improved the base editing efficiency and exhibited a high product purity in zebrafish. The gene-editing system provides not only a tool for gene function analysis in zebrafish, but also a new platform for modeling human diseases, in addition to drug discovery and assessment of toxic agents or medicine.

Although this tool will greatly promote the development of precise base-editing technology in zebrafish, there are still several important issues needed to be noticed. One of the key issues is the off-target effects. Recently, several groups found that CBEs can generate off-target single-nucleotide variations in both DNA and RNA [21–23]. Meanwhile, several BE variants with reduced RNA or even DNA binding capacity have been developed to reduce such off-target activities [21, 22]. Using these improved BEs, the number of off-target DNA and RNA editing sites could be reduced to basal levels, which is similar to the level in the control cells. Off-target RNA is probably not a problem in zebrafish and can be avoided to transmit in the next generation by breeding edited male with a wild-type female. Zebrafish can produce large numbers of progeny; it should be relatively easy to identify the true mutants. These advantages make gene editing a wide application in zebrafish.

Another issue concerned is the target window. Our results demonstrated that the zAncBE4max system has the same targeting window as the BE3 system (positions 3–7) in zebrafish. We know that many of the human genetic diseases are caused by single point mutation. Several base editors have been developed for modeling human diseases with the mutation. BE-PLUS, a base editor by fusing 10 copies of GCN4 peptide to nCas9 (D10A) for recruiting scFv-APOBECUGI-GB1 to the target sites, has an enlarged targeting window (positions 4–16) [24]. By using diversified lamprey cytidine deaminases and various fusing strategies, a base editing tool can target the frontward (C-PmCDA1-BE, positions − 1 to 6) and backward (C-8-BE, positions 9–14) regions of the target window [25]. Obviously, replacing the Spcas9 using different variants is still an alternative way to enlarge the targeting scope in the genome, which has been proved to work efficiently in many model species [26, 27]. Further optimization using these strategies will greatly improve the editing ability in zebrafish.

## Conclusions

Overall, the engineered zAncBE4max provides a promising tool for zebrafish model establishment and enriches the genome editing toolbox in zebrafish.

## Methods

### Zebrafish husbandry and breeding

Wild-type AB fish zebrafish (*Danio rerio*) were purchased from the Institute of Hydrobiology of the Chinese Academy of Sciences (Wuhan, China). Zebrafish strains were raised and maintained at 28.5 °C in a water recirculation system under a cycle of 14 h:10 h light/dark. The development of embryos was staged by standard morphological criteria [28].

### Construction optimization and synthesis

The coding sequence used in the zAncBE4max system was optimized by changing base usage following zebrafish codon preference and taking into account both the GC content and secondary mRNA structure, and synthesized by GenScript based on AncBE4max sequence [16]. To obtain pCMV-zAncBE4max, the full length of the zebrafish codon-optimized sequence was cloned into a pCMV vector (Addgene #112094). The pCMV-zAncBE4max plasmid was linearized by HindIII and used as a template for in vitro transcription. The capped mRNA was synthesized using the mMessage mMachine T7 Ultra Kit (Ambion AM1345, Austin, TX, USA). All gRNAs templates were prepared according to the cloning-independent gRNA generation method [29]. gRNAs were transcribed in vitro by using the T7 RNA Polymerase Systems (Thermo Fisher Scientific, Grand Island, NY, USA). All RNAs are purified by the RNeasy Mini Kit (Qiagen 74104, Germany). All oligos were listed in Additional file 2: Table S4. BE3 plasmid (pCMV-BE-zCas9) was a gift from Professor Shuo Lin (Addgene # 101739).

### Embryo microinjection, Sanger sequencing, and genotyping

zAncBE4max mRNA (300 ng/μl) and gRNA (30 ng/μl) were mixed and co-injected into zebrafish embryos of one-cell stage. Injected embryos were incubated at 28.5 °C. Embryos that developed normally at 48 hpf were collected in groups (6 embryos in one group). Genomic DNA was isolated using the NaOH-based extraction method. Targeted genomic loci were amplified from genomic DNA and then cloned into the pGEM®-T Easy Vector System I (A1360, Promega, Madison, WI, USA) for sequencing. To identify germline-transmitted mutations, the injected founder embryos (F0) were raised to adulthood and then crossed with wild types to generate heterozygous embryos (F1). Homozygotes were produced by incross of heterozygotes. Genotyping was performed by PCR amplification of target sites using caudal fin DNA and sequencing.

### Base editing efficiency and deep sequencing

Genomic DNA from a pool of 100 injected embryos for a target was isolated by a routine method. The genomic region covering the target was PCR-amplified (Additional file 2: Table S4). An amplicon library was constructed for each target and sequenced using the MGISEQ2000 sequencing platform. After sequencing, the raw data were analyzed using the Soapnuke software to obtain clean reads [30]. Sequence comparison and alignment with wild-type sequences were performed to obtain information on base change and indel. Three injection repeats and sequencing were performed for each target.

### Quantitative real-time PCR

Total RNAs were isolated from whole embryos (30 embryos per group) at 48 hpf, using TRIzol Reagent (15596-026, Thermo Fisher, Grand Island, NY, USA) following the manufacturer’s protocol. The cDNA was reverse transcribed from 1 μg total RNAs using MMLV (M1701, Promega, Madison, WI, USA). SYBR Green qPCR Mix (D01010, GeneCopoeia, Rockville, MD, USA) was used for quantitative real-time PCR amplification in a Step One real-time PCR system (Applied Biosystems, USA). All quantitative PCR was performed in triplicates.

### Imaging and video recording

Zebrafish embryos were anesthetized with 0.03% tricaine (Sigma-Aldrich, USA) and mounted in 4% methylcellulose. Photographs were taken by a Zeiss Axio Imager Z1 microscope and processed by the Adobe Photoshop CC software. The animal video was recorded by Optronis (CR600x2) and processed by Adobe After Effects and Apowersoft video converter studio.

### Statistical analysis

All data were presented as means ± standard error of the mean from at least three independent experiments. Statistical comparisons were made using the Mann-Whitney *U* test. Statistics analysis was performed using the GraphPad Prism 6 software package (GraphPad Software, La Jolla, USA). *p* < 0.01 was considered to be statistically significant.

## Supplementary Information

**Additional file 1: Fig.S1.** Whole zebrafish codon-optimized zAncBE4max and its alignment with AncBE4max sequence (nucleotides and amino acids). **Fig. S2.** Indel frequency (%).

**Additional file 2: Table S1.** Germline transmission rate. **Table S2.** Sequencing data. **Table S3.** Original values related to Fig. 3. **Table S4.** Primer sequences and PCR conditions.

**Additional file 3: Movie 1.** *twist2* E78K heterozygous mutants and WT zebrafish at 6 months.

## Data Availability

All data generated or analyzed during this study are included in this published article, its supplementary information files, and publicly available repositories. Sequence data have been deposited in GenBank under BioProject accession number PRJNA674464 (https://www.ncbi.nlm.nih.gov/bioproject/PRJNA674464). The generated raw reads have been deposited in the NCBI Sequence Read Archive (SRA) (http://www.ncbi.nlm.nih.gov/sra) under accession numbers SRR12999158~SRR12999193. Raw data can be found in Additional file 2: Table S3.
