# Supplementary figures and images for: An optimized base editor with efficient C-to-T base editing in zebrafish
Source: BMC Biol. 2020 Dec 3;18:190. doi: 10.1186/s12915-020-00923-z (PMC7716464; doi:10.1186/s12915-020-00923-z)

Fig. S2.

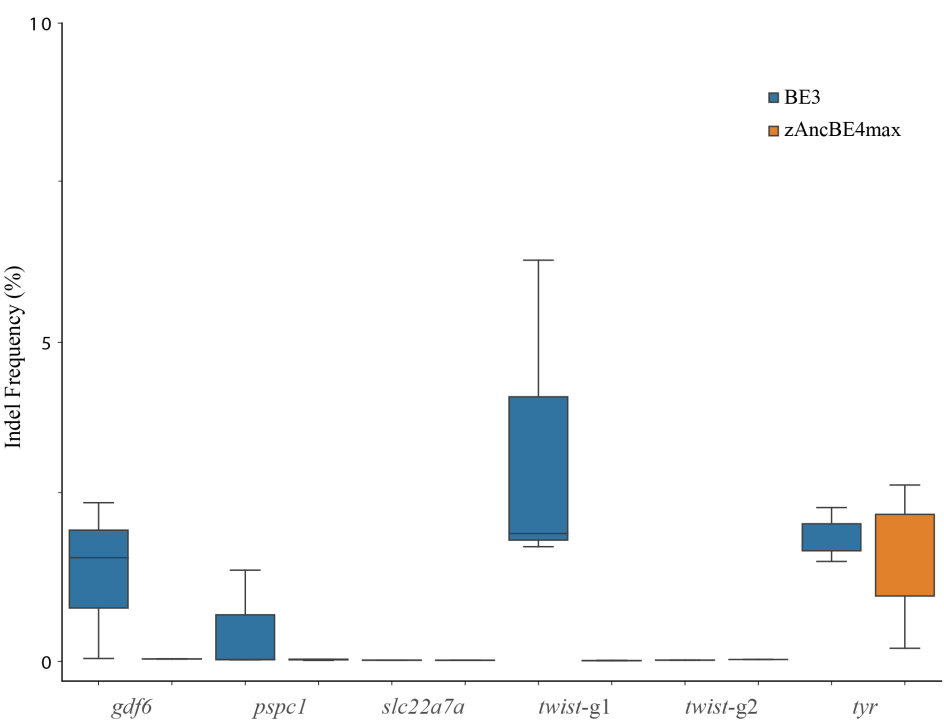

Supplement: Supplementary file 1 — Additional file 1: Fig.S1. Whole zebrafish codon-optimized zAncBE4max and its alignment with AncBE4max sequence (nucleotides and amino acids). Fig. S2. Indel frequency (%). [file 12915_2020_923_MOESM1_ESM.zip › Fig S2.pdf]
