# Supplementary material for: An optimized base editor with efficient C-to-T base editing in zebrafish
Source: BMC Biol. 2020 Dec 3;18:190. doi: 10.1186/s12915-020-00923-z (PMC7716464; doi:10.1186/s12915-020-00923-z)
Supplement: Supplementary file 2 — Additional file 2: Table S1. Germline transmission rate. Table S2. Sequencing data. Table S3. Original values related to Fig. 3. Table S4. Primer sequences and PCR conditions. [file 12915_2020_923_MOESM2_ESM.zip › Table S1.pdf]

Table S1. Germline transmission rate

Gene	Base Editor	Germline Targeting Efficiency
tyr	BE3	16.67% (3/18)
	zAncBE4max	43.75% (7/16)
twist2-g1	BE3	21.43% (3/14)
	zAncBE4max	52.94% (9/17)
twist2-g2	BE3	12.50% (2/16)
	zAncBE4max	41.18% (7/17)
slc22a7a	BE3	28.57% (4/14)
	zAncBE4max	62.50% (10/16)
pspc1	BE3	18.75% (3/16)
	zAncBE4max	46.15% (6/13)
gdf6	BE3	23.53% (4/17)
	zAncBE4max	57.14% (8/14)
