# Supplementary material for: An optimized base editor with efficient C-to-T base editing in zebrafish
Source: BMC Biol. 2020 Dec 3;18:190. doi: 10.1186/s12915-020-00923-z (PMC7716464; doi:10.1186/s12915-020-00923-z)
Supplement: Supplementary file 2 — Additional file 2: Table S1. Germline transmission rate. Table S2. Sequencing data. Table S3. Original values related to Fig. 3. Table S4. Primer sequences and PCR conditions. [file 12915_2020_923_MOESM2_ESM.zip › Table S2.pdf]

Table S2. Sequencing data

**Number of reads**	***gdf6***	***pspc1***	***slc22a7a***	***twist2*-g1**	***twist2*-g2**	***tyr***
BE3	1,581,483	255,730	14,760,976	118,427	24,564,283	1,499,927
zAncBE4max	669,401	263,304	17,709,073	372,904	22,493,231	1,016,578
