# Supplementary material for: An optimized base editor with efficient C-to-T base editing in zebrafish
Source: BMC Biol. 2020 Dec 3;18:190. doi: 10.1186/s12915-020-00923-z (PMC7716464; doi:10.1186/s12915-020-00923-z)
Supplement: Supplementary file 2 — Additional file 2: Table S1. Germline transmission rate. Table S2. Sequencing data. Table S3. Original values related to Fig. 3. Table S4. Primer sequences and PCR conditions. [file 12915_2020_923_MOESM2_ESM.zip › Table S4.pdf]

Table S4. Primer sequences and PCR conditions

Genes/fragments	GenBank access No.	Primer sequence (5'-3')	Tm (°C)
tyr	NM_131013.3	F: ATTTTGTGCTAATGTCGTTCACT<br>R: CGTTATGCAGCTCAAATCTCG	55
twist2-g1	NM_001005956.2	F: AGTTCTAGCTCTCCCGTCT<br>R: GTTGTCCATCTCGTCGCTCT	55
twist2-g2	NM_001005956.2	F: GTTGTCCATCTCGTCGCTCT<br>R: CCGAGCTCCGTCAATAAACGTA	56
Slc22a7a	NM_001083861.1	F: TTTATACATCCTATGGCTTCCG<br>R: TGGCACAGACACATTTGACC	57
pspc1	NM_001045258.1	F: AAACAGACCCATTCGTATTC<br>R: ATACAGATTCATGCAAAAAAACA	55
gdf6	NM_001159994.1	F: CTCCTTTGCGAAGACAAACGTA<br>R: ACCAAGTTGCTTTAGGTCGAT	55
twist2	NM_001005956.2	F: GCTCTCTCCTTTGCGAAGAC<br>R: AATCCCCCAAACGTCCAGAA	57
runx2a	NM_212858.2	F: GACCTCACGACAACCGGACCA<br>R: TCCAGCCATTACCGTCACCAC	60
runx2b	NM_212862	F: TTCTCTGCTCGGTTTTGCCTT<br>R: GTCCCACAAAGCGCAGGTCA	58
osx	NM_212863.2	F: CTCAATCCTCAAATGCCGAGT<br>R: GTATCCAGAACCACGAGCTGA	57
osc	NM_001110123.1	F: ACATCTATTATGCGTCTCAACCC<br>R: CCAGTTCCTTAATGGCGTCT	56

alp	NM_201007.2	F: AAATGAAGGTCGTACAACTGC<br>R: AGTTGTGTTTCCTCTCCGTTC	55
col1a2	NM_182968	F: GCATCATGTCAATCGGGCCTC<br>R: TCAGGTCCTTTAGCGCCAT	57
bmp2a	NM_131359.1	F: CACAGACGGACACCCTAACCA<br>R: AGCCTACGTCGCTGAAGTCCA	58
bmp2b	NM_131360.2	F: CGCTTGCTCAATATGTTCGGAT<br>R: ACTTCGTATCGTGTTTGCTCT	57
Il-1 β	NM_212844.2	F: GCACATCAAACCCCAATCCAC<br>R: ATACTGATCGCAAATCGTGCAT	58
tnfa	NM_212859.2	F: TGCTTCACGCTCCATAAGACC<br>R: AGCTGATGTGCAAAGACACC	55
